# Antiretroviral-Mediated Microglial Activation Involves Dysregulated Autophagy and Lysosomal Dysfunction

**DOI:** 10.3390/cells8101168

**Published:** 2019-09-28

**Authors:** Ashutosh Tripathi, Annadurai Thangaraj, Ernest T. Chivero, Palsamy Periyasamy, Shannon Callen, Maria E. Burkovetskaya, Ming-Lei Guo, Shilpa Buch

**Affiliations:** Department of Pharmacology and Experimental Neuroscience, 985880 Nebraska Medical Center, University of Nebraska Medical Center, Omaha, NE 68198, USA; ashutosh.tripathi@unmc.edu (A.T.); annadurai.thangaraj@unmc.edu (A.T.); ernest.chivero@unmc.edu (E.T.C.); palsamy.periyasamy@unmc.edu (P.P.); scallen@unmc.edu (S.C.); mburkovetskaya@unmc.edu (M.E.B.)

**Keywords:** combined antiretroviral therapy, lysosome, autophagy, microglial activation, neuroinflammation

## Abstract

In the era of combined antiretroviral therapy (cART), as infected individuals continue to have longer lifespans, there is also an increased prevalence of HIV-associated neurocognitive disorders (HAND). Inflammation is one of the underlying features of HAND, with the role of viral proteins and antiretroviral drugs implicated in this process. Microglia are extremely sensitive to a plethora of stimuli, including viral products and cART. The current study was undertaken to understand the molecular mechanism(s) underlying cART-mediated activation of microglia. Herein we chose a combination of three commonly used drugs, tenofovir disoproxil fumarate (TDF), emtricitabine (FTC), and dolutegravir (DTG). We demonstrated that exposure of microglia to this cART cocktail induced lysosomal membrane permeabilization (LMP), which subsequently resulted in impaired lysosomal functioning involving elevated pH and decreased cathepsin D (CTSD) activity. cART exposure of microglia resulted in increased formation of autophagosomes as demonstrated by a time-dependent increase of autophagy markers, with a concomitant defect in the fusion of the lysosomes with the autophagosome. Taken together, our findings suggest a novel mechanism by which cART impairs lysosomal functioning, resulting in dysregulated autophagy and increased neuroinflammation. Interventions aimed at lysosome protection could likely be envisioned as promising therapeutic targets for abrogating cART-mediated microglia activation, which in turn, could thus be considered as adjunctive therapeutics for the treatment of HAND pathogenesis.

## 1. Introduction

In the era of combined antiretroviral therapy (cART) as infected individuals continue to enjoy longer lifespans, HIV infection has been transformed from a death sentence to a more chronic and manageable disease [1,2,3]. Paradoxically, however, with increasing lifespans, these individuals also have an increased prevalence of varying degrees of neurocognitive decline collectively termed as HIV-associated neurocognitive disorders (HAND), which severely impacts quality of life. Almost 50% of infected individuals develop HAND, ranging from asymptomatic to mild cognitive-motor disorders [3]. In fact, epidemiological studies have demonstrated “accelerated aging” in HIV+ individuals on cART [4,5]. Inflammation, mediated by both viral proteins and cART, has been implicated as a significant underlying factor for the pathogenesis of HAND [6,7]. Of note, there is evidence of persistent neuroinflammation in cART-treated HIV+ individuals; the precise mechanism(s) underlying increased neuroinflammation, however, remain less understood [2,8,9].

Microglia, the resident macrophages of the CNS accounting for about 10% to 15% of all brain cells, are tightly regulated and can dynamically alter their function in response to inflammatory stimuli [10,11]. Following external stimulation, microglia undergo morphological changes and/or proliferation resulting in the production and secretion of a plethora of cytokines, chemokines, and neurotoxic factors. These secreted factors can, in turn, impact neighboring neurons, resulting in neuronal excitotoxicity and damage [12,13]. Activation of microglia can yield two outcomes: Moderate activation, which plays a role in CNS homeostasis via immunological surveillance, or prolonged activation-mediated neuroinflammation, which can lead to significant neuronal dysfunction and cognitive impairment, resulting in exacerbated damage within the CNS. While the activation of microglia by HIV-1 Tat has been extensively studied [14,15,16,17,18], there are also reports on cART-mediated activation of microglia in vitro [19,20]; however, very little is known in regards to mechanisms underlying cART-mediated activation of microglia.

Lysosomes are specialized cellular organelles that mediate protein degradation via various pathways, such as endocytosis, phagocytosis, or autophagy. Most of the damaged or misfolded proteins are degraded by endogenous lysosomal enzymes followed by the recycling of their products to provide nutrients/energy for cell survival and growth [21]. Autophagy is an evolutionarily conserved process by which cytoplasmic components are sequestered in double membrane vesicles, known as autophagosomes, which can fuse directly with lysosomes to form autolysosomes [22]. This enables delivery of the autophagic cargo to the autolysosomes where these materials are degraded by acidic lysosomal hydrolases. Functional lysosome is required for autophagy clearance [23,24,25]. Lysosome storage disorders are caused by abnormal lysosomal function, leading, in turn, to accumulation of undegraded metabolites [21,26]. Most lysosome storage disorders are characterized by underlying inflammation and autophagy dysregulation. In the central nervous system, impairment of lysosomal function has been shown to result in microglial activation and increased neuroinflammation [27,28].

Herein, we sought to explore cART-mediated activation of microglia and the possible involvement of lysosomal dysfunction-mediated autophagy dysregulation as an underlying mechanism(s). We acknowledge that there are several combinations of antiretroviral drugs or cART that are approved clinically by the established guidelines for use in adults and adolescents living with HIV-1 [29]. In the present study, we chose to use a combination of tenofovir disoproxil fumarate (TDF), emtricitabine (FTC), and dolutegravir (DTG). The rationale for this combination is as follows. It is well-recognized that the first-line therapy for HIV-1 infection comprises of two nucleoside reverse transcriptase inhibitors (NRTIs) plus a boosted protease inhibitor or an integrase inhibitor [30]. This combinatorial therapy achieves significant plasma viral suppression in the host (lower than 50 copies/mL). Based on this, we chose to use two reverse transcriptase inhibitors (TDF and FTC) as well as an integrase inhibitor (DTG) as the cART cocktail for our current study. This combination has also been effectively used in the clinical setting [31,32,33,34].

In the present study, cART-mediated lysosomal dysfunction and its role in microglial activation was assessed. Our data demonstrates the cART cocktail induced lysosomal membrane permeabilization (LMP), impaired lysosomal functioning (elevated pH), decreased cathepsin D (CTSD) activity, and increased formation of autophagosomes, with a concomitant defect in the fusion of the lysosomes with the autophagosome. Thus, our findings establish a link between defective autophagy and lysosomal dysfunction, which in turn, contributes to microglial activation in response to cART exposure and strongly suggest that lysosomal dysfunction plays a critical role in the pathogenesis of HAND in HIV-infected individuals on cART, and that lysosome protection could be considered as an adjunctive therapeutic strategy for ameliorating/dampening some of the neurological complications of HAND.

## 2. Materials and Methods

### 2.1. Reagents

Antiretroviral drugs TDF and FTC (Gilead Sciences, Foster City, CA, USA), and DTG (ViiV Healthcare, Research Triangle Park, NC, USA). Rapamycin (R8781) and bafilomycin A1 (B1793) were purchased from Sigma-Aldrich, St. Louis, MO, USA. Antibody resources: BECN1 (sc-11427) and CTSB (sc-365558) were purchased from Santa Cruz Biotechnology, Dallas, TX, USA. LAMP2 (NB300-591) and MAP1LC3B (NB100-2220) were purchased from Novus Biological Company, Centennial, CO, USA. CTSD (ab75852) and M6PR (ab124767) were purchased from Abcam, Cambridge, MA, USA. TFEB (A303-673A) was purchased from Bethyl Laboratories, Montgomery, TX, USA. SQSTM1 (MBL PM045) was purchased from MBL International, Woburn, MA, USA. Goat anti-rabbit (sc-2004) and goat anti-mouse (sc-2005) were purchased from Santa Cruz Biotechnology, Dallas, TX, USA. 

### 2.2. Rat Primary Microglial Cell Isolation

Rat primary microglial cells (rPMs) from either sex were isolated from Sprague-Dawley newborn pups (1–3 days). After digestion and dissociation of the dissected brain cortices in Hank’s buffered salt solution (HBSS, Thermo Fisher Scientific Waltham, MA, USA, 14025076) supplemented with 0.25% trypsin (Thermo Fisher Scientific Waltham, MA, USA, 25300-054), mixed glial cultures were prepared by re-suspending the cell suspension in Dulbecco’s Modified Eagle Medium (DMEM, Thermo Fisher Scientific Waltham, MA, USA, 11995-065) supplemented with 10% heated inactivated fetal bovine serum (FBS, Thermo Fisher Scientific Waltham, MA, USA; 16000-044) with 100 U/mL penicillin, and 0.1 mg/mL streptomycin. Cells were plated at a 20 × 10^6^ cells/flask density onto 75-cm^2^ cell culture flasks. Cell medium was replaced every 3 days, and after the first medium change, macrophage colony-stimulating factor (Thermo Fisher Scientific Waltham, MA, USA, PHC9504) at 0.25 ng/mL was added to the flasks to promote microglial proliferation. When confluent (7–10 days), mixed glial cultures were subjected to shaking at 37 °C at 220 g for 2 h, to promote microglia detachment from the flasks. The cell medium, containing the released microglia cells, was collected from each flask and centrifuged at 1000 *g* for 5 min to collect cells, then plated on cell culture plates for all subsequent experiments. The purity of microglial cultures was evaluated by immune-histochemical staining using the antibody specific for AIF1 (Wako Pure Chemical Industries, Chuo-ku, Osaka, Japan, 019-19741) and was >95% pure.

### 2.3. Antiretroviral Treatment

Antiretroviral stock solutions were prepared by dissolving the drugs (TDF, FTC, and DTG) in dimethyl sulfoxide (DMSO). Final concentrations of each antiretroviral drug (TDF, FTC, and DTG) in the cART cocktail were 5 μM. The final concentration of DMSO was less than 0.01% (*v*/*v*) in the cAR- treated and control groups.

### 2.4. Western Blotting

Treated cells were lysed using the mammalian Cell Lysis kit (Sigma-Aldrich, St. Louis, MO, USA, MCL1-1KT). Equal amounts of the proteins were electrophoresed in a sodium dodecyl sulfate-polyacrylamide gel (12%) under reducing conditions followed by transfer to polyvinylidene difluoride (PVDF) membranes (Sigma-Aldrich, St. Louis, MO, USA, IPVH00010). The membranes were blocked with 5% nonfat dry milk in 1× Tween-Tris-buffered saline (TTBS, 1.21 g Tris [Fisher Scientific, Hampton, NH, USA, BP152-5], 8.77 g NaCl [Fisher Scientific, Hampton, NH, USA, BP358-212], 500 μL Tween-20 [Fisher Scientific, Hampton, NH, USA, BP337-500], pH 7.6 for 1 L). Western blots were then probed with antibodies recognizing the indicated proteins. The loading protein amounts were normalized by actin beta (ACTB, Sigma-Aldrich, St. Louis, MO, USA, A5441). The secondary antibodies were HRP conjugated to goat anti-mouse/rabbit IgG.

### 2.5. Immunocytochemistry

For immunocytochemistry, rPMs were plated on coverslips. The next day, cells were fixed with 4% paraformaldehyde for 15 min at room temperature, followed by permeabilization with 0.3% Triton X-100 (Fisher Scientific, Hampton, NH, USA, BP151-500) in phosphate-buffered saline (PBS, Fisher Scientific, Hampton, NH, USA, SH3025801). Cells were then incubated with a blocking buffer containing 10% normal goat serum in PBS for 1 h at room temperature followed by addition of primary antibodies and incubated overnight at 4 °C. Finally, the secondary antibodies were added for 2 h. Next, cells were washed 3 times in PBS and mounted with Prolong gold antifade reagent with 4,6-diamidino-2-phenylindole (DAPI, Thermo Fisher Scientific, Waltham, MA, USA, P36935). Fluorescence images were taken with a Zeiss Observer using a Z1 inverted microscope (Carl Zeiss, Thornwood, NY, USA), and the acquired images were analyzed using the Axio Vs 40 Version 4.8.0.0 software (Carl Zeiss, Thornwood, NY, USA).

### 2.6. Quantification of MAP1LC3B and LAMP2 Puncta

Fluorescence images were taken with a Zeiss Observer using a Z1 inverted microscope (Carl Zeiss, Thornwood, NY, USA) and the acquired images were analyzed using Image J software. Firstly, the region of interest or the cells to be analyzed were selected using the polygon selection tool. The green channel was extracted to grayscale followed by photographic inversion (GFP fluorescence converted to black pixels over a white background). The regions of interest to be measured were then analyzed by the measure particles algorithm to record the GFP-LC3 puncta number, area, and size. Results were displayed in the results window and were transferred to an excel spreadsheet using the functions of ImageJ.

### 2.7. Real-Time qPCR

Total RNA was extracted using a Quick-RNA™ Miniprep Kit (Zymo Research, Irvine, CA, USA, R1055) as per the manufacturer’s protocol. Column purified total RNA was then reverse transcribed into cDNA using a Verso cDNA Synthesis Kit (Thermo Fisher Scientific, Waltham, MA, USA, AB-1453/B), according to the manufacturer’s instructions. Reverse transcribed RNA was then successively analyzed by the 7500 Fast Real-Time PCR System (Applied Biosystems, Grand Island, NY, USA). Real-time PCR was performed using the TaqMan Fast Advanced Master mix and TaqMan gene expression assays (Applied Biosystems) with FAM-labeled probes using standard amplification protocol. Rat primers for *Tnf (Rn01525859_g1), Il6* (Rn01410330_m1)*, Il1b* (Rn00580432_m1), *Ccl2* (Rn00580555_m1), *Il10* (Rn01483988_g1), *Il4* (Rn01456866_m1), *Lamp2* (Rn00567053_m1), *Ctsd* (Rn00592528_m1), and *Gapgh* (Rn01775763_g1) were purchased from Applied Biosystems. Normalization was done with *Gapdh*, an internal control. Each reaction was carried out in triplicate, and three independent experiments were run. The fold change in expression was then obtained by the 2^−ΔΔCT^ method.

### 2.8. CTSD Activity Determination

CTSD activity was measured using the CTSD Activity Assay Kit (Fluorometric) from Abcam Cambridge, MA, USA (ab65302). Cell lysates were collected for analysis. The cell lysate was incubated with reaction buffer for 1 h at 37 °C. Fluorescence was measured (Ex/Em = 328/460 nm). Fold increases in proteases activity were determined by comparing the relative fluorescence units (RFUs) against the levels of the controls.

### 2.9. Lysosomal Membrane Permeability Assay

Acridine orange is a fluorescent dye that easily traverses the cell membrane. It is a weak base, which reversibly accumulates into acidified membrane-bound compartments. The fluorescence emission of acridine orange is concentration-dependent, from red at high concentrations (e.g., in lysosomes) to green at low concentrations (e.g., in the cytosol), with yellow as intermediate (e.g., upon trapping in nucleoli). The shift in the red-to-green emission ratio in comparison to controls may thus either monitor lysosomal leakage or change in lysosomal pH. In our fluorometric studies, cells were cultured in 96-well culture plates. Cells were first exposed with acridine orange (5 μg/mL) at 37 °C for 15 min. Cells were rinsed, then incubated in HBSS with or without cART for the indicated times. Cells were examined at 1-h intervals using a Synergy™ Mx Monochromator-Based Multi-Mode Microplate Reader (BioTek Instruments, Inc. Winooski, VT, USA) with the excitation wavelength at 485 nm and emission recorded at 530 and 620 nm.

### 2.10. Lysosomal pH Measurement

Lysosomal pH was measured using LysoSensor (Yellow/Blue DND-160) (Thermo Fisher Scientific, Waltham, MA, USA)—a dual ratio-metric indicator dye that is used to measure the pH of acidic organelles, such as lysosomes. Briefly, rPMs were incubated with 2 μM LysoSensor for 5 min at 37 °C and fluoresecence intensity recorded at 340 and 380 nm, following which 340/380 nm ratios were converted to pH units using a calibration curve established using 20 mM MES (+120 mM KCl, 20 mM NaCl, 10 µM Monensin, 20 µM Nigericin) and the pH was adjusted between 3.0 and 7.0 using either HCl or NaOH.

### 2.11. MAP1LC3B Turnover and SQSTM1 Degradation Assays

The methodology for MAP1LC3B turnover and SQSTM1 degradation assays has already been published [16,35]. Briefly, rPMs were seeded at a density of 5 × 10^5^ cells/well in a 6-well plate. The plates were then incubated in a humidified 5% CO_2_ incubator at 37 °C for attachment. Cells were then starved overnight in the serum-free culture medium. rPMs were then exposed to either cART (5 µM each of TDF, FTC, and DTG) for 24 h alone or the cells also exposed to 400 nM bafilomycin (BAF) in the last 4 h of the 24 h cART treatment. At the end of the experiment, cells were harvested, and protein samples were prepared for western blotting analysis. 

### 2.12. Plasmids Transfection

The procedure for transfection of both plasmids; tandem fluorescent-tagged MAP1LC3B plasmid (ptfLC3; a gift from Tamotsu Yoshimori; Addgene, 21074) [36] and pEGFP HSPA plasmid (a gift from Lois Greene; Addgene, 15215) [37] were similar. Briefly, cells were maintained with 10% FBS DMEM overnight. At 70% confluence, the culture medium was replaced with 250 µL of Opti-MEM^®^ I Reduced Serum Medium. Cells were transfected with the GFP-MAP1LC3B plasmid using Lipofectamine^®^ 3000 Reagent, according to the manufacturer’s protocol, for 12 h, following which the culture medium was replaced with the respective 10% heat-inactivated FBS-DMEM for 24 h. Then, cells were treated with various reagents for the indicated time and processed for further analysis.

### 2.13. Statistical Analysis

The results are presented as means ± SEM and were evaluated using a one-way analysis of variance followed by a Bonferroni (Dunn) comparison of groups using least squares-adjusted means. Probability levels of <0.05 were considered statistically significant.

## 3. Results

### 3.1. cART-Mediated Impairment of Lysosomal Function in rPMs

**Figure 1:**
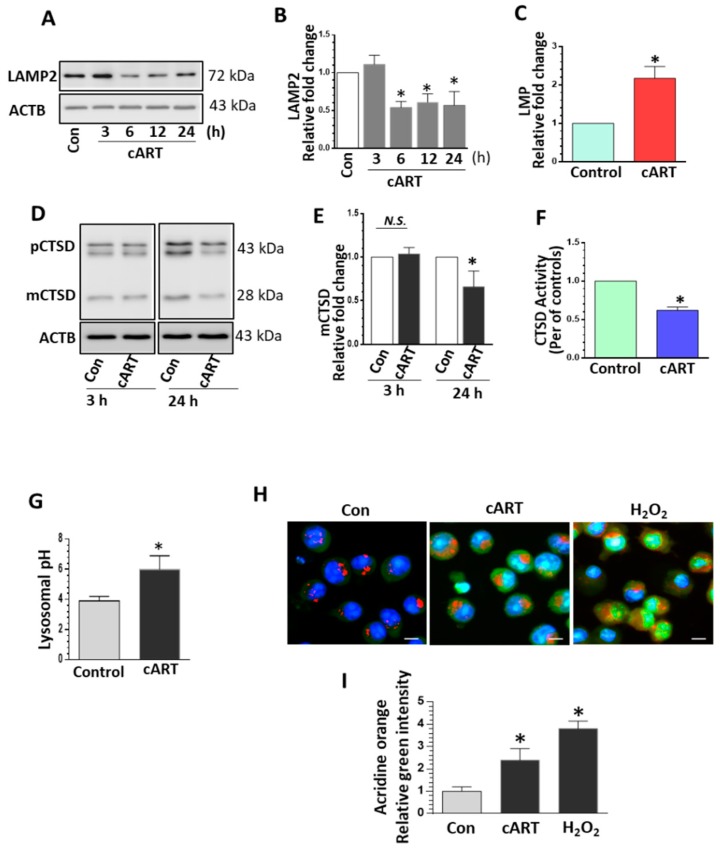
Exposure of rat primary microglial cells (rPMs) to combined antiretroviral therapy (cART) cocktail resulted in impaired lysosomal function. rPMs were seeded into six-well plates and treated with cART (5 µM each of tenofovir disoproxil fumarate (TDF), emtricitabine (FTC), and dolutegravir (DTG)) for the indicated time periods. (**A**,**B**) Exposure of microglia to cART resulted in a significant decrease in expression of lysosomal-associated membrane protein 2 (LAMP2) at 6 to 24 h post-treatment. (**C**) Representative bar graph showing cART-mediated significantly increased lysosomal membrane permeabilization (LMP) (24 h). (**D**,**E**) Microglia exposed to cART demonstrated a significant decrease in levels of mature cathepsin D (mCTSD) at 24 h post-treatment. (**F**) Representative bar graph showing exposure of cART significantly reduced the CTSD activity in rPMs (24 h). (**G**) Representative bar graph showing cART-mediated increased lysosomal pH in rPMs. (**H**,**I**) Acridine orange staining showing increased green color and reduced red color in cART-treated rPMs. H_2_O_2_ was used as a positive control for lysosome damage. Data is from three independent experiments. Actin beta (ACTB) served as a protein loading control for western blots. Data are expressed as means ± SEM and were analyzed using student t-test or one-way ANOVA. *, *p* < 0.05 vs. control; N.S., non-significant.

The lysosome is a critical cellular organelle responsible for clearing cytosolic debris. It has been shown that impairment of lysosomal function resulted in microglial activation and increased neuroinflammation in the CNS [27,28]. We first sought to determine the effects of the three antiretrovirals (DTG, TDF, FTC) individually and in every combination on lysosomal function. rPMs were exposed to cART (TDF, FTC, DTG), each at 5 µM for 24 h. The rationale for choosing these concentrations of drugs is based on several published reports [38,39,40]. Patient studies have reported levels of antiretrovirals in CSF to be 32 ng/mL for TDF, 386 ng/mL for FTC, and 23 ng/mL for DTG [38]. In a recent in vitro study, differential accumulation of TDF, FTC, and DTG was reported in various CNS cells, including microglia, when the cells were treated with 5 or 10 µM concentrations of these drugs [39]. In the current study, adverse effects of TDF, FTC, and DTG (individual and in combinations) on lysosomal proteins (decreased lysosomal-associated membrane protein 2 (LAMP2) and mature cathepsin D (mCTSD) expression) as well as lysosomal functions (increased LMP and decreased CTSD activity) were observed (Supplementary Figure S1A–D). Interestingly, the most adverse effects of cART on lysosomes were observed in cells exposed to the combination of three antiretroviral drugs (often recommended as a first-line therapy for HIV-1 infection) [30]. Moreover, we checked the toxicity of the cART cocktail by analyzing cell survival. As shown in Supplementary Figure S1E, there is no significant difference in the cell survival of the cART-treated and non-treated rPMs. Thus, the combination of three antiretrovirals (cART), TDF, FTC, and DTG (each at 5 µM), was chosen for the subsequent experiments. Next, the rPMs were exposed to cART for 3 to 24 h. The protein homogenates of the treated cells were assessed for the expression of the lysosomal marker, LAMP2. As shown in Figure 1A, in rPMs exposed to cART, there were significant downregulation of LAMP2 expression starting at 6 h, with a continued trend of downregulation up to 24 h. As LAMP2 is the lysosome membrane protein, its downregulation could affect membrane permeability. Furthermore, we checked cART-mediated LMP in rPMs. Exposure of rPMs with cART for 24 h significantly increased LMP (Figure 1C). Next, we sought to examine cART-mediated effects on the expression of lysosomal cathepsins. Cathepsins can be divided into three groups: Cysteine, aspartic, and serine proteases. CTSD is an aspartic protease while CTSB is a cysteine protease [41,42,43]. Interestingly, cART exposure resulted in decreased expression of mature cathepsin D (mCTSD) at 24 h in rPMs exposed to cART (Figure 1D,E). Next, we also examined the expression of yet another cathepsin—Cathepsin B (CTSB). As shown in Figure S2A, exposure of cART significantly downregulated the expression of CTSB in rPMs at 24 h. We next sought to examine CTSD activity to check the lysosomal functioning in the context of cART. There was a significant decrease in CTSD activity in rPMs exposed to cART for 24 h (Figure 1F). Maturation of cathepsins and its activity is dependent on the acidity of the lysosome (low pH) [41,42,43]. Next, we sought to determine the lysosome pH in the rPMs treated with cART. Exposure of cART increased the lysosomal pH in rPMs (Figure 1G). We also performed acridine orange staining in rPMs exposed to cART to further validate the findings observed with LMP. Acridine orange is a fluorescent dye that easily traverses the cell membrane. Being a weak base, acridine orange reversibly accumulates in acidified membrane-bound compartments, such as the lysosomes. Furthermore, the fluorescent emission of acridine orange is concentration dependent, being red at high concentrations (e.g., in lysosomes) to green at low concentrations (e.g., in the cytosol), with yellow as an intermediate (e.g., upon trapping in nucleoli). Thus, either lysosomal leakage or lysosomal pH change could be easily monitored by determining shifts in the red-to-green emission ratio in comparison with the respective control cells. As shown in Figure 1H,I, rPMs exposed to cART exhibited increased green emission. 

Next, we also sought to determine cART-mediated transcriptional regulation and trafficking of lysosomal proteins. CTSD as well as other soluble lysosomal proteins are transported to the lysosomes via mannose-6-phosphate receptor (M6PR)-mediated trafficking [44]. There was no notable change in the expression of M6PR in the rPMs treated with cART for varying time periods (Figure S2B). Transcription factor EB (TFEB) plays key role in lysosome biogenesis [45]. rPMs were exposed to cART for varying time periods (3, 6, 12, and 24 h), and the protein expression levels of TFEB were determined by western blotting. As shown in Figure S2C, there was no significant change in the expression of TFEB in cART-exposed rPMs. Next, we examined mRNA levels of *Lamp2* and *Ctsd* in rPMs exposed to cART for various time periods. As shown in Figure S2D,E, there were no significant changes in the mRNA expression of TFEB target genes of the lysosome pathway (*Lamp2* and *Ctsd)*. The results suggest that cART directly affects the lysosome and there are no effects on trafficking and transcriptional regulation of lysosome-associated proteins.

### 3.2. cART-Mediated Dysregulated Autophagy in rPMs

**Figure 2:**
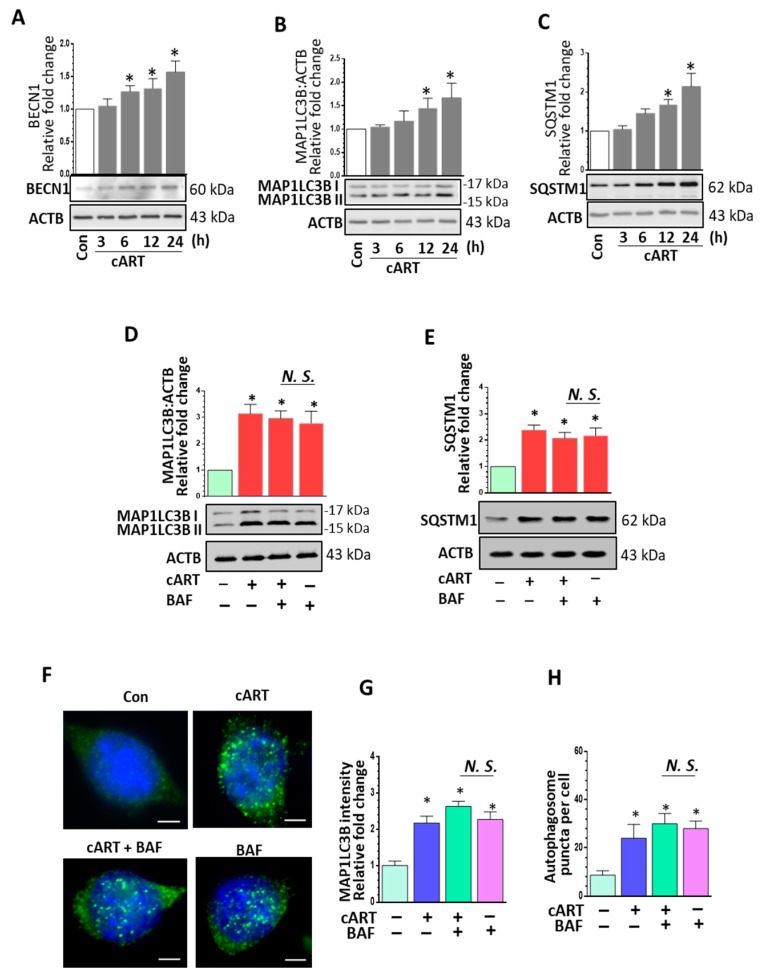
Exposure of rPMs to cART resulted in dysregulated autophagy. (**A**–**C**) Representative western blots and bar graphs showing cART-mediated increased expression levels of autophagy markers beclin 1 (BECN1), microtubule-associated protein 1 light chain 3 beta (MAP1LC3B), and sequestosome 1 (SQSTM1) in rPMs. (**D**,**E**) Representative western blots and bar graph showing MAP1LC3B lipidation and SQSTM1 accumulation in rPMs treated with cART, bafilomycin (BAF, autophagosome fusion inhibitor), and cART + BAF. (**F**) Representative fluorescent-microscopic image showing cART-mediated increased MAP1LC3B puncta (autophagosomes). (**G**,**H**) Representative bar graphs showing cART-mediated increased intensity of MAP1LC3B and increased numbers of MAP1LC3B puncta. Data is from three independent experiments. ACTB served as a protein loading control for western blots. Data are expressed as means ± SEM and were analyzed using student t-test or one-way ANOVA. *, *p* < 0.05 vs. control; N.S., non-significant.

Based on the premise that during normal physiological processes lysosomes fuse with autophagosomes to form autolysosomes to ensure completion of autophagy and clearance of misfolded proteins, we thus rationalized that impairment of lysosomal function could likely perturb this process, resulting in blockage of autophagic flux. We next sought to assess the effects of cART on autophagy mediators. We determined the expression of multiple autophagy-related proteins, including BECN1/Beclin1 (beclin 1, autophagy related), MAP1LC3B/LC3B (microtubule-associated protein 1 light chain 3 beta), and SQSTM1/p62 (sequestosome 1), in rPMs treated with cART. As shown in Figure 2A–C, expression of BECN1, MAP1LC3B, and SQSTM1 was significantly upregulated in a time-dependent manner. These results thus implicated that the action of cART on the lysosomes involved blockage of the autophagy maturation stage. To assess whether the increased quantity of MAP1LC3B was a result of enhanced autophagosome synthesis or reduced autophagosome turnover (due to delayed trafficking or reduced fusion with the lysosomes), we assessed MAP1LC3B lipidation and SQSTM1 expression in the presence of bafilomycin A_1_ (BAF—a known inhibitor of autophagosome fusion) alone, cART alone, and a combination of BAF with cART. MAP1LC3B-II levels are thus directly correlated with the number of autophagosomes [46]. It has been reported that impaired degradation and accumulation of SQSTM1 protein is directly correlated with the rate of autophagic vesicle degradation [47]. As shown in Figure 2D,E, western blot analysis showed no significant difference in the accumulation of MAP1LC3B-II and SQSTM1 in rPMs exposed to cART in the presence or absence of BAF. Immunofluorescence imaging also showed increased expression and number of MAP1LC3B puncta in rPMs exposed to cART (Figure 2F–H). Overall, the result shows dysregulated autophagy and accumulation of autophagosomes in the cART-treated rPMs.

### 3.3. cART-Mediated Impairment of Autophagosome–Lysosome Fusion

**Figure 3:**
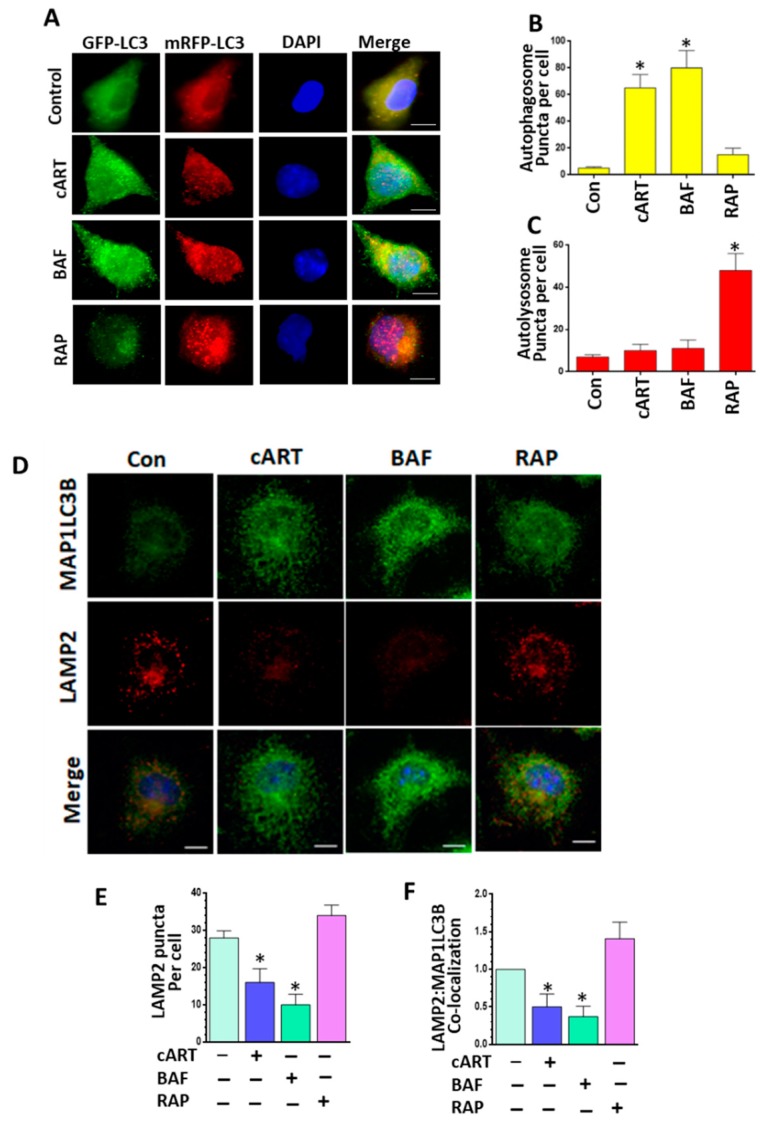
Exposure of microglia to cART resulted in blockade of autophagosome–lysosome fusion. (**A**) rPMs were seeded into a 12-well plate followed by tandem fluorescent-tagged MAP1LC3B plasmid. Next, cells were exposed to cART (5 µM each of TDF, FTC, and DTG) for an additional 24 h and observed by confocal imaging. The results showed that cART exposure significantly increased the formation of autophagosomes (yellow puncta). (**B**) Representative bar graph showing the number of autophagosome (yellow puncta) per cell. (**C**) Representative bar graph showing the number of autolysosome (red puncta) per cell. (**D**) rPMs were seeded into 12-well plates followed with cART exposure for 24 h. Cells were then double immunostained with MAP1LC3B and LAMP2 antibody and observed by immunofluorescent microscopy. (**E**,**F**) Representative bar graphs showing cART-mediated decreased LAMP2 puncta and decreased colocalization of MAP1LC3B and LAMP2. BAF—autophagosome fusion inhibitor, and rapamycin (RAP—autophagy inducer) were used as controls for autophagy flux. Data is from three independent experiments and is expressed as means ± SEM and were analyzed using one-way ANOVA. *, *p* < 0.05 vs. control.

Based on our biochemical studies that cART interrupts the maturation stage of autophagy by impairing lysosomal functioning, we next sought to validate this observation further. For this, rPMs were first transfected with tandem fluorescent-tagged MAP1LC3B plasmid and then exposed to cART followed by an assessment of the autophagic flux. The degree of autophagic flux in rPMs is reflected by the distribution pattern (wide distribution or puncta formation) as well as by the fluorescent color (yellow or red) [36]. Under basal conditions, both green and red signals are evenly distributed throughout the cells. During autophagosome formation, there is increased formation of yellow puncta due to colocalization of green and red fluorescence; in the maturation stage, on the other hand, GFP is unstable in the autolysosomes due to the internal acidic environment, leading to quenching of the green fluorescence with the presence of only the red puncta. As shown in Figure 3A–C**,** cART significantly increased the yellow puncta in rPMs. This effect was analogous to the treatment of cells with BAF, a well-known inhibitor of autophagosome–lysosome fusion and was used here as a positive control. Exposure of transfected cells to rapamycin (RAP) on the other hand, a known autophagy inducer, resulted in a strikingly increased presence of red puncta in rPMs, thereby indicating an increased fusion of the autophagosome with the lysosome. Next, we sought to investigate the effect of cART on endogenous autophagosome–lysosome fusion. For this, rPMs were exposed to cART for 24 h followed by double staining of cells with MAP1LC3B as well as the lysosomal marker LAMP2. As shown in Figure 3D–F, under basal conditions, MAP1LC3B (green signal) was evenly distributed throughout the cells with some colocalization with lysosomes (yellow signal). Following cART exposure, however, there was increased intensity of MAP1LC3B with a concomitant decrease of LAMP2 signal, thereby indicating decreased lysosomal colocalization.

### 3.4. cART-Mediated Activation of rPMs

**Figure 4:**
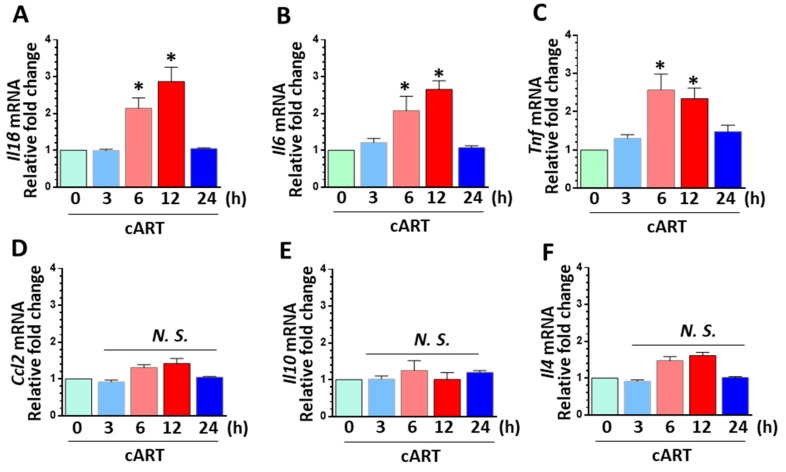
Exposure of microglial cells to cART resulted in cellular activation. rPMs were seeded into six-well plates and treated with cART (5 µM each of TDF, FTC, and DTG) for the indicated time periods. Total RNAs were extracted for the detection of pro- and anti-inflammatory mediators. (**A**–**C**) Representative bar graphs showing cART-mediated time-dependent increase in the mRNA expression of pro-inflammatory cytokines interleukin 1 beta (Il1b), interleukin 6 (Il6), and tumor necrosis factor (Tnf) in rPMs. (**D**–**F**) cART exposure had no effect on the expression of C-C motif chemokine ligand 2 (Ccl2) and anti-inflammatory cytokines (Il10 and Il4) in rPMs. Data is from three independent experiments and is represented as means ± SEM using one-way ANOVA. *, *p* < 0.05 vs. control.

Moderate activation of microglia plays a role in CNS homeostasis via immunological surveillance; however, prolonged activation mediates neuroinflammation. To explore the effects of cART on microglial activation, rPMs were either left untreated or were exposed to cART (TDF, FTC, DTG), each at 5 µM for 3 to 24 h. To determine cART-mediated microglial activation, we assessed proinflammatory cytokine levels in rPMs. Increased levels of proinflammatory cytokines are the determinant of microglial activation. We sought to determine the expression levels of various pro- and anti-inflammatory mediators in the control and cART-treated rPMs. For that, total RNA was extracted from the cells for the detection of various pro- and anti-inflammatory mediators by qPCR. Our findings demonstrated that cART time-dependently increased the mRNA levels of Il1b (interleukin 1 beta), with a significant up-regulation at 6 and 12 h post-treatment (Figure 4A). A similar trend was observed for the expression of proinflammatory cytokines, Il6 (interleukin 6; Figure 4B) and Tnf (tumor necrosis factor; Figure 4C). There was no notable change in the expression of CCL2 (C-C motif chemokine ligand 2) expression in the presence of cART (Figure 4D). Additionally, we also assessed the expression of two anti-inflammatory mediators in cells treated with cART and found no significant difference in the expression of Il10 (interleukin 10) and Il4 (interleukin 4) in rPMs exposed to cART in a time-dependent manner (Figure 4E,F).

### 3.5. HSPA Overexpression Abrogated cART-Mediated Impairment of Lysosomal Function

**Figure 5:**
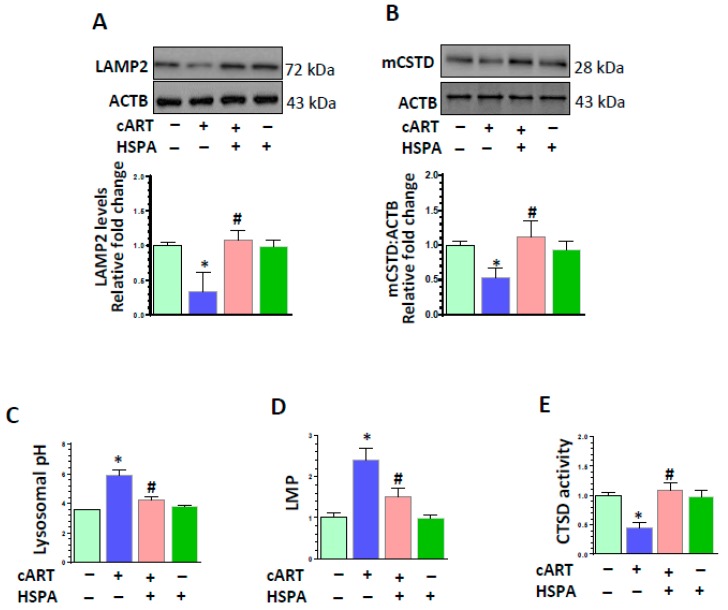
HSPA overexpression abrogated cART-mediated impairment of lysosomal function. Control rPMs and heat shock protein family A (HSPA) overexpressing rPMs were seeded into six-well plates subjected to various treatments for 24 h. Protein homogenates were prepared for the detection of the indicated molecules. (**A**,**B**) Representative western blots showing overexpressing HSPA in rPMs reversed cART-mediated downregulation of LAMP2 and mCTSD expression levels. (**C**) Representative bar graph showing overexpression of HSPA in rPMs protected lysosomal pH. (**D**,**E**) Representative bar graphs showing HSPA protected LMP (**D**), and CTSD activity (**E**) in cART-treated rPMs. For all western blots, ACTB served as a protein loading control. Data is from three independent experiments and is expressed as means ± SEM and were analyzed using student t-test or one-way ANOVA. *, *p* < 0.05 vs. control; ^#^, *p* < 0.05 vs. cART.

Having determined the importance of LMP in cART-mediated lysosomal dysfunction, we next sought to determine the protective role of HSPA (heat shock protein family A) in blocking LMP [48,49] in cART-exposed microglial cells. First, we have checked the expression levels of HSPA in the rPMs treated with cART in a time-dependent manner. As shown in Supplementary Figure S3A, there was no significant difference in the expression of HSPA after cART exposure in rPMs. As it is well known that HSPA protects the lysosome membrane in stress conditions [48,49], we next sought to investigate the protective effects of HSPA overexpression in cART-treated rPMs. First, we overexpressed the HSPA in rPMs (Figure S3B). Overexpression of HSPA in rPMs protected lysosomal function after cART treatment. As shown in Figure 5, HSPA overexpression abrogated cART-mediated downregulation of LAMP2 (Figure 5A) and mCTSD (Figure 5B) in rPMs. Along with this, we also checked the pH of the lysosome. HSPA overexpression protected the acidity of the lysosome in the presence of cART (Figure 5C). Additionally, as shown in Figure 5D,E, HSPA overexpression in rPMs significantly abrogated cART-mediated upregulation of LMP with concomitant downregulation of CTSD activity.

### 3.6. HSPA Overexpression Abrogated cART-Mediated Autophagy Dysregulation and Microglial Activation

**Figure 6:**
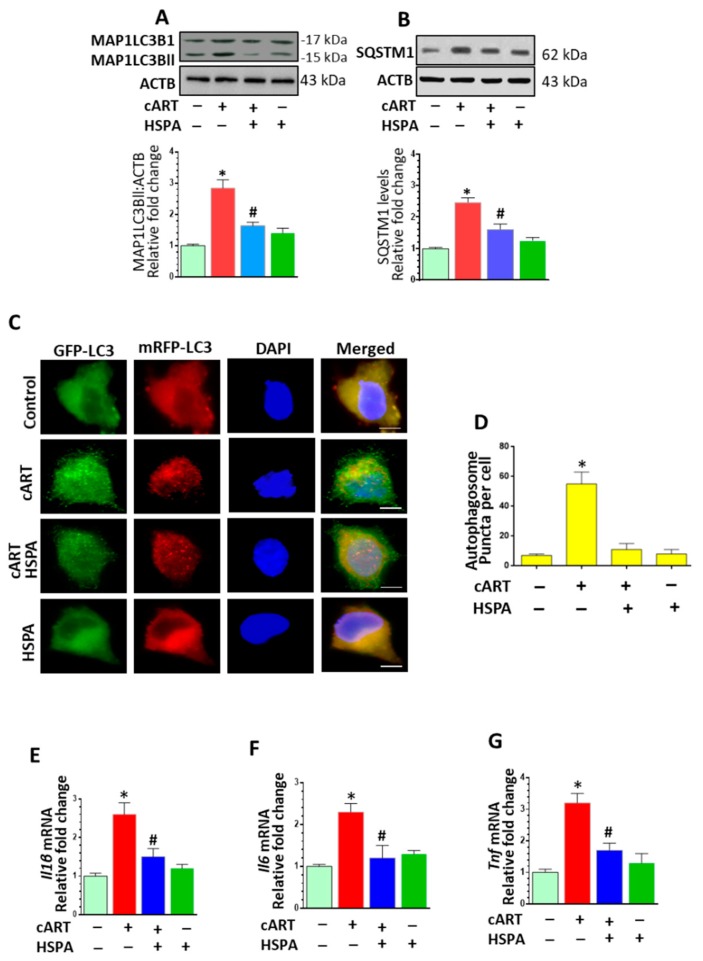
HSPA overexpression abrogated cART-mediated autophagy dysregulation and microglia activation. (**A**,**B**) Representative western blots and bar graphs showing overexpression of HSPA in rPMs reversed cART-mediated upregulation of autophagy markers MAP1LC3B and SQSTM1. (**C**,**D**) HSPA-overexpressing rPMs were seeded into 12-well plates followed with the transfection of tandem fluorescent-tagged MAP1LC3B plasmid. Then, cells were exposed to various treatments for another 24 h and fluorescent intensity was assessed by confocal microscopy. HSPA reversed cART-mediated induction of autophagosome formation. (**E**–**G**) Representative bar graphs showing HSPA overexpression abrogated cART-mediated increase in the mRNA expression of pro-inflammatory cytokines Il1b, Il6, and Tnf in rPMs. For all western blots, ACTB served as a protein loading control. Data is from three independent experiments and is expressed as means ± SEM and was analyzed using student t-test or one-way ANOVA. *, *p* < 0.05 vs. control; ^#^, *p* < 0.05 vs. cART.

The next step was to explore the protective effects of HSPA on cART-mediated dysregulation of autophagy. As expected, and as shown in Figure 6A,B, HSPA overexpression in rPMs notably blocked cART-mediated upregulation of MAP1LC3B and SQSTM1, thereby implying increased autophagosome–lysosome fusion. To validate these findings and to decipher the ability of cART to regulate the autophagosome–lysosome fusion efficiency, HSPA-overexpressing rPMs were transfected with a plasmid-encoding tandem fluorescent-tagged MAP1LC3B followed by exposure of cells to cART for 24 h. As shown in Figure 6C,D, HSPA-overexpressing rPMs transfected with the tandem fluorescent-tagged MAP1LC3B reporter plasmid and exposed to cART exhibited a significant increase in the yellow puncta with a concomitant decrease in the red puncta, thereby indicating incomplete autophagosome maturation. However, HSPA overexpression in rPMs followed by cART exposure (24 h) demonstrated a significant increase in the red puncta with moderate levels of yellow puncta compared with only cART-exposed rPMs. We next sought to examine whether HSPA overexpression could also block cART-mediated activation of microglia. As shown in Figure 6E–G, HSPA overexpression in rPMs significantly blocked cART-mediated upregulation of pro-inflammatory cytokine (Il1β, Il6, and Tnf) mRNA.

## 4. Discussion

Microglial activation and increased neuroinflammation are hallmark features of HAND pathogenesis in HIV-infected individuals on cART [2,8,9]. The advent of cART has resulted in the successful suppression of peripheral viremia; paradoxically, however, emerging evidence has suggested that long-term use of cART could result in severe side effects, including oxidative stress, DNA and mitochondrial damage, and disruption of phagocytosis in different cells [50,51,52]. As is well-recognized, there are several combinations of antiretroviral drugs that are clinically used to treat HIV infection [29]. In the present study, we demonstrated that exposure of microglia to the combination of three commonly used ARVs (TDF, FTC, DTG) mediated impaired lysosomal functioning, leading, in turn, to blockade of autophagosome–lysosome fusion, ultimately resulting in microglial activation.

CNS inflammation is a common underlying feature in HIV-infected patients on cART [53]. Multiple factors have been suggested to contribute to the increased neuroinflammation: (a) The persistent expression of HIV proteins, such as Tat and gp120; (b) low levels of HIV replication in brain macrophages owing to reduced penetrance of some cART regimens; (c) co-morbid conditions, including hepatitis C co-infection and substance abuse; and (d) direct CNS toxicity of cART. While sufficient information is present on viral protein-mediated inflammation, the role of cART as a contributor of neuroinflammation specifically via its action on glial cells, such as microglia, is now garnering attention. Our findings demonstrated that cART-mediated upregulation of pro-inflammatory mediators, such as Il6, Il1b, and Tnf mRNA levels. Furthermore, mRNA analysis of anti-inflammatory markers (Ccl2, Il10, and Il4) showed no significant changes in rPMs exposed to cART, suggesting thereby cART mediated skewing of the microglia towards a pro-inflammatory phenotype.

Autophagy dysregulation and neuroinflammation are closely related in the development of neurodegeneration [54]. Autophagy dysregulation is a hallmark of HIV neuropathogenesis [55,56]. Previous reports have shown dysregulated autophagy in the prefrontal cortex of postmortem brains of persons with HIV-1-associated encephalitis [57]. Another study also demonstrated disruption of neuronal autophagy by SIV-infected microglia, which resulted in neurodegeneration [58]. Since dysregulated autophagy and inflammation is believed to be a “driving force” underlying pathogenesis of HAND, our results implied that cART could contribute to promotion and exaggeration of HAND. There are few important studies demonstrating antiretroviral-mediated autophagy dysregulation in different cell types. EFV has been shown to induce autophagy and aberrant differentiation in normal human keratinocytes [59] and mitophagy in hepatic cells [60]. Zidovudine, another NRTI, has been shown to adversely affect mitochondrial turnover in primary T cells [61]. Other protein inhibitors, such as atazanavir, have also been shown to induce autophagy and mitophagy in human preadipocytes [62] while saquinavir has been demonstrated to upregulate endoplasmic reticulum stress, autophagy, and apoptosis in ovarian cancer cells [63]. Various etiologies have been suggested for antiretroviral-mediated autophagy dysregulation and cellular damage; however, the mechanism(s) regulating autophagy dysregulation have not been conclusively explored.

Our findings also demonstrated cART mediated dysregulation of autophagy as assessed by increased expression of autophagy mediators BECN1, MAP1LC3B, and SQSTM1. The MAP1LC3B turnover assay, as well as the SQSTM1 degradation assay, also indicated blockage of the autophagic flux as evidenced by the accumulation of MAP1LC3B-II and SQSTM1 in cART-exposed rPMs. The addition of BAF (autophagosome–lysosome fusion inhibitor) to cART-treated rPMs was unable to cause any further increase in the expression of MAP1LC3B-II and SQSTM1, which confirms maximal accumulation of MAP1LC3B-II and SQSTM1 by cART in rPMs. In vitro assessment of autophagic flux using tandem fluorescent-tagged MAP1LC3B plasmid also demonstrated cART-mediated blockage of autophagy, by increased accumulation of autophagosomes but not autolysosomes.

It is well known that lysosomal dysfunction causes autophagosome accumulation [21,23]. Lysosomes are specialized cellular organelles critical for the maturation stage of autophagy since the fusion of autophagosomes with lysosomes to form the autolysosome is necessary for protein degradation [64,65]. Lysosomes are a target of HIV proteins, and lysosomal dysfunction is inherently involved in HAND pathogenesis [66]. The lysosomal membrane is composed of highly glycosylated transmembrane proteins, such as LAMP, which are thought to protect the membrane from degradation by lysosomal enzymes. LMP is the major cause of lysosomal pH elevation. Protons leak through the destabilized membrane, resulting in a loss of the pH gradient [67,68]. Our results showed cART mediated downregulation of LAMP2 expression levels and increased LMP. Cathepsins are stable and active in the acidic environment (low pH) of the lysosomes [41,42,43]. Results from our data showed that there is downregulation of mCTSD expression and decreased CTSD activity, with concomitant increase in lysosomal pH in the rPMs treated with cART. Overall, cART-mediated LMP causes pH elevation, which in turn leads to decreased CTSD activity. Additionally, the effects of cART on cathepsin regulation and trafficking were also determined in this study. It is well documented that cathepsins, as well as other soluble lysosomal proteins, are transported into the lysosomes via M6PR-mediated trafficking [44]. In this study, exposure of cART to rPMs failed to alter the expression levels of M6PR protein. Next, we determined the expression of TFEB, a master regulator of the lysosome pathway. Interestingly, there was no significant change in the expression of TFEB in cART-treated rPMs. Moreover, there was no change in the mRNA expression levels of Lamp2 and Ctsd. Overall, this confirms that cART directly affect the lysosome and there is no cART-mediated transcriptional regulation of lysosome-associated proteins.

Having determined the importance of LMP in cART-mediated lysosomal dysfunction in rPMs, we next sought to protect the lysosome membrane to reverse cART-mediated lysosome and autophagy defects. It has been well documented that HSPA localizes to the luminal side of the endosomal-lysosomal system and stabilizes the lysosome membrane to protect cells from LMP against various stimuli [48,49]. Our findings demonstrated that overexpression of HSPA in rPMs significantly abrogated cART-mediated lysosomal dysfunction (evidenced by increased LAMP2, mCTSD, and pH, and decreased LMP), autophagy dysregulation (evidenced by decreased MAPLC3BII and p62), and microglia activation (evidenced by a significant reduction in pro-inflammatory cytokines). These observations thus imply that LMP-mediated lysosomal dysfunction lies upstream of the autophagy dysregulation. These results further suggested that strategies to protect lysosome could help to dampen cART-mediated inflammation in HIV-infected individuals on cART.

**Figure 7:**
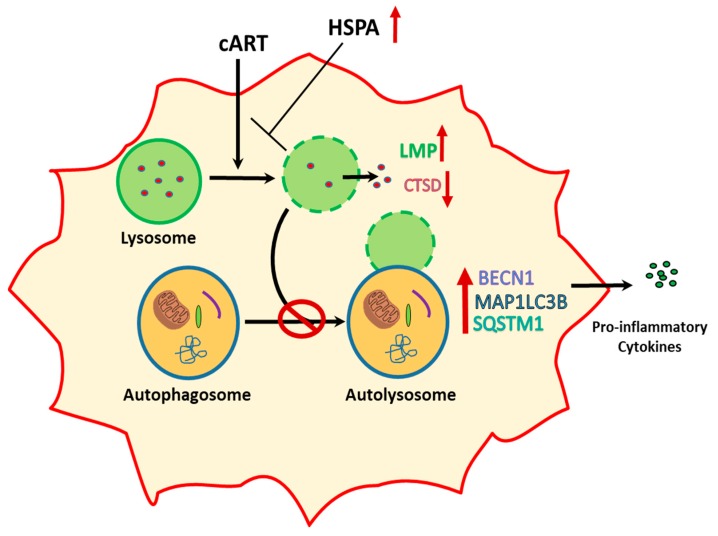
Schematic depicting cART-mediated activation of microglia involving lysosomal and autophagy dysregulation and the ability of HSPA to protect LMP to reverse these effects of cART.

In summary, our findings demonstrated that LMP-mediated lysosome dysfunction is central in cART-mediated autophagy dysfunction and microglial activation and the lysosome membrane protection reversed cART-mediated effects (Figure 7). Lysosomal protection agents could thus be developed as future adjunctive treatment options for dampening cART-mediated inflammation in HIV-infected individuals on cART.

## Supplementary Materials

The following are available online at https://www.mdpi.com/2073-4409/8/10/1168/s1, Figure S1: Effects of individual and combinations of antiretrovirals (tenofovir disoproxil fumarate (TDF), emtricitabine (FTC), and dolutegravir (DTG)) on lysosome pathway in rPMs. Figure S2: Effects of cART on mannose-6-phosphate receptor (M6PR), transcription factor EB (TFEB) and TFEB target genes of lysosome pathway. Figure S2: Effect of cART on heat shock protein family A (HSPA) expression levels.

## Abbreviations

ACTB	actin, beta
BAF	bafilomycin A1
BECN1	beclin 1, autophagy related
cART	combined antiretroviral therapy
CCL2	C-C motif chemokine ligand 2
CNS	central nervous system
CTSD	cathepsin D
CTSB	cathepsin B
DAPI	4,6-Diamidino-2-phenylindole
DMEM	Dulbecco modified eagle medium
DTG	dolutegravir
FBS	fetal bovine serum
FTC	emtricitabine
GAPDH	glyceraldehyde-3-phosphate dehydrogenase
HAND	HIV-1-associated neurocognitive disorders
HIV-1 Tat	human immunodeficiency virus-1 transactivator of transcription
IL1B	interleukin 1, beta
IL6	interleukin 6
LAMP2	lysosome associated membrane protein 2
LMP	lysosomal membrane permeabilization
M6PR	mannose-6-phosphate receptor
MAP1LC3B	microtubule-associated protein 1 light chain 3 beta
NRTIs	nucleoside reverse transcriptase inhibitors
PBS	phosphate buffered saline
RAP	rapamycin
rPMs	rat primary microglial cells
SQSTM1	sequestosome 1
TFEB	transcription factor EB
TDF	tenofovir disoproxil fumarate
TNF	tumor necrosis factor
