# Supplementary material for: Antiretroviral-Mediated Microglial Activation Involves Dysregulated Autophagy and Lysosomal Dysfunction
Source: Cells. 2019 Sep 28;8(10):1168. doi: 10.3390/cells8101168 (PMC6829395; doi:10.3390/cells8101168)
Supplement: Supplementary file 1 [file cells-08-01168-s001.zip › cells-600233-supplementary.pptx]

## Slide 1
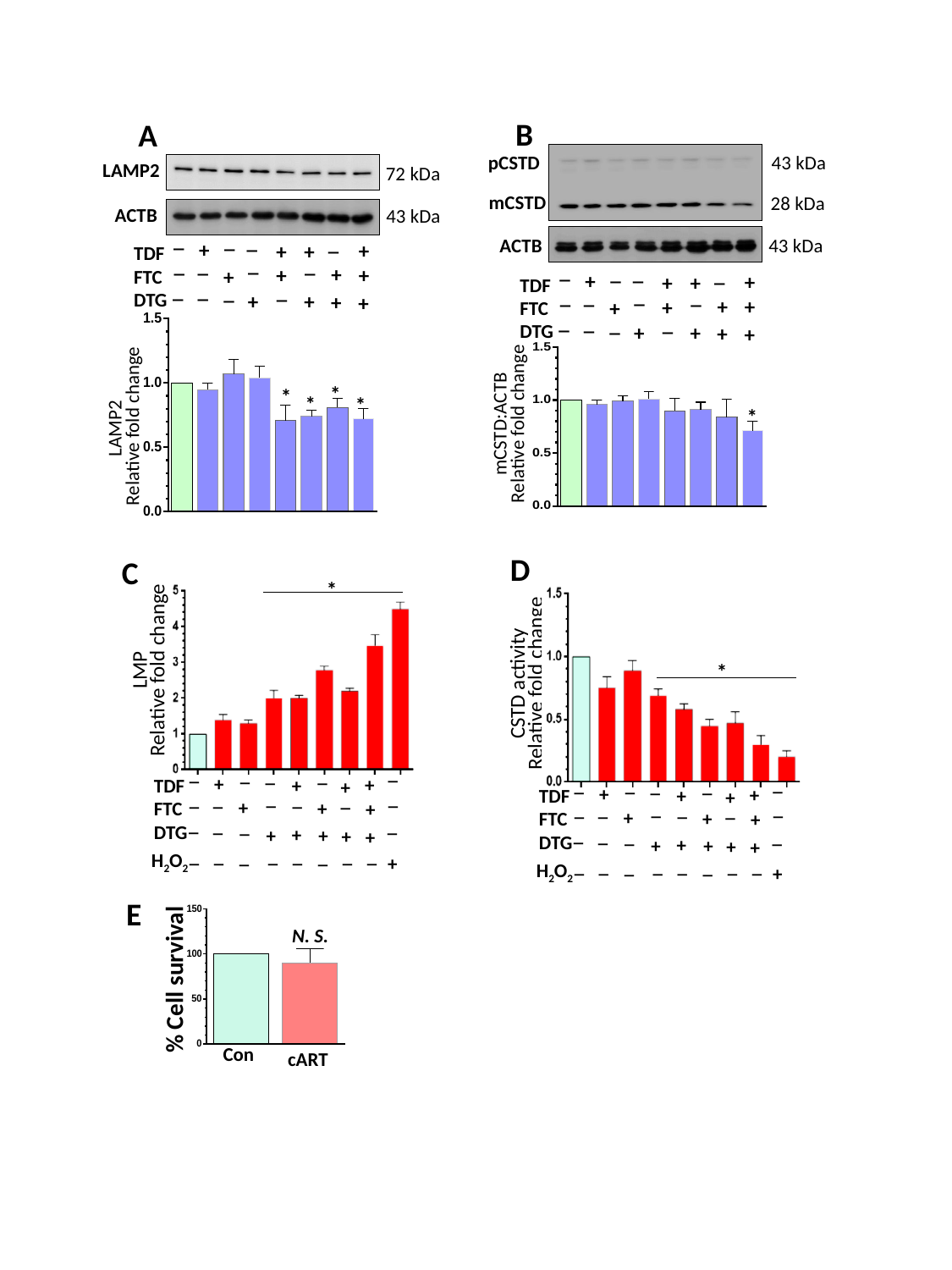

B
A
43 kDa
pCSTD
mCSTD
ACTB
_
_
_
_
+
+
+
+
TDF
FTC
DTG
_
_
_
_
+
+
+
+
_
_
_
_
+
+
+
+
LAMP2
72 kDa
28 kDa
ACTB
43 kDa
_
_
_
_
+
+
+
+
TDF
FTC
DTG
_
_
_
_
+
+
+
+
_
_
_
_
+
+
+
+
43 kDa
*
*
*
*
*
LAMP2
Relative fold change
mCSTD:ACTB
Relative fold change
D
*
CSTD activity
Relative fold change
_
_
_
_
_
+
+
TDF
FTC
DTG
+
+
_
_
_
_
_
_
+
+
+
_
_
_
_
+
+
+
+
+
_
_
_
_
_
_
_
_
H2O2
+
C
*
LMP
Relative fold change
_
_
_
_
_
+
+
TDF
FTC
DTG
+
+
_
_
_
_
_
_
+
+
+
_
_
_
_
+
+
+
+
+
_
_
_
_
_
_
_
_
H2O2
+
E
% Cell survival
Con
cART
N. S.

## Slide 2
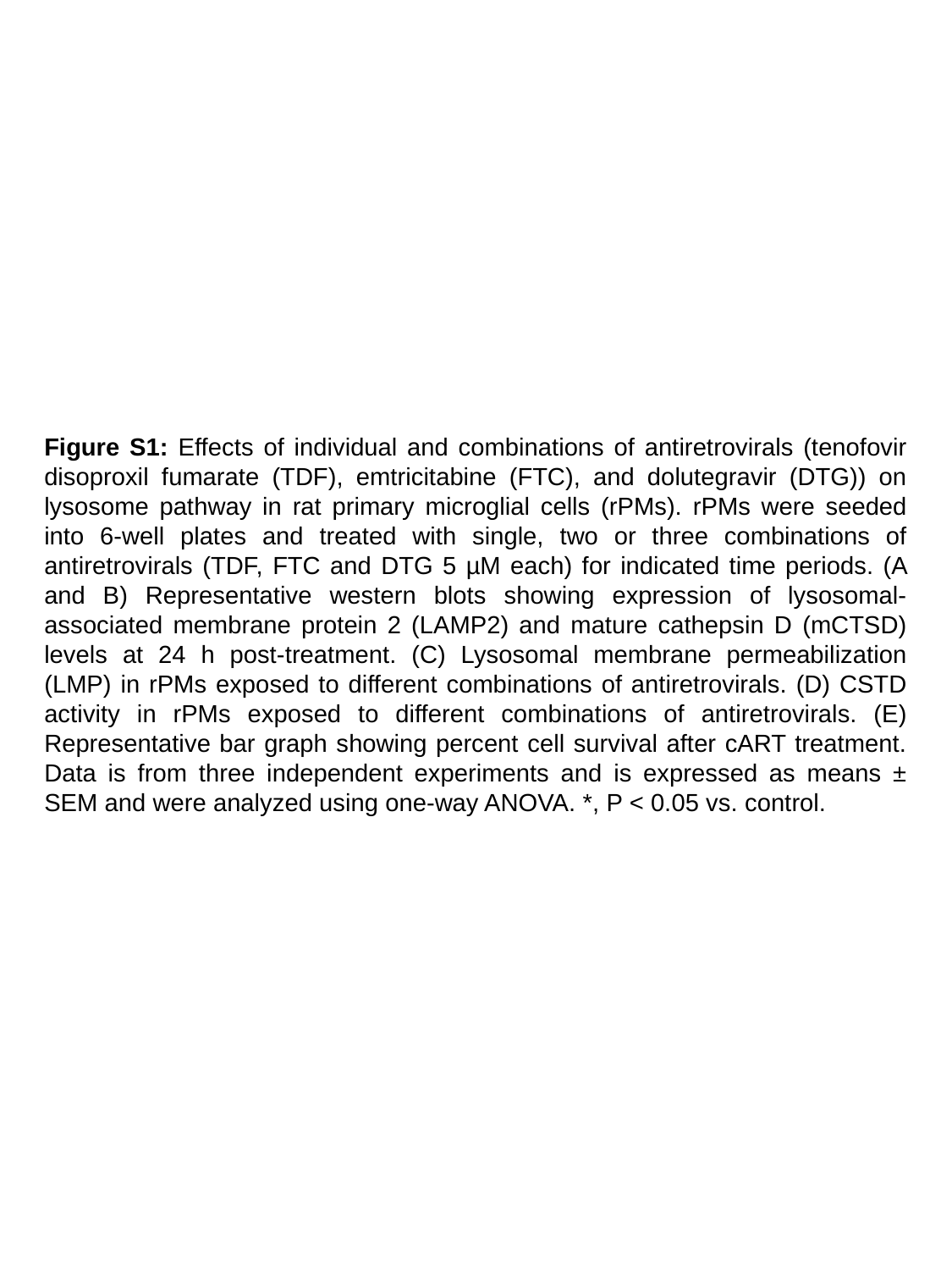

Figure S1: Effects of individual and combinations of antiretrovirals (tenofovir disoproxil fumarate (TDF), emtricitabine (FTC), and dolutegravir (DTG)) on lysosome pathway in rat primary microglial cells (rPMs). rPMs were seeded into 6-well plates and treated with single, two or three combinations of antiretrovirals (TDF, FTC and DTG 5 µM each) for indicated time periods. (A and B) Representative western blots showing expression of lysosomal-associated membrane protein 2 (LAMP2) and mature cathepsin D (mCTSD) levels at 24 h post-treatment. (C) Lysosomal membrane permeabilization (LMP) in rPMs exposed to different combinations of antiretrovirals. (D) CSTD activity in rPMs exposed to different combinations of antiretrovirals. (E) Representative bar graph showing percent cell survival after cART treatment. Data is from three independent experiments and is expressed as means ± SEM and were analyzed using one-way ANOVA. *, P < 0.05 vs. control.

## Slide 3
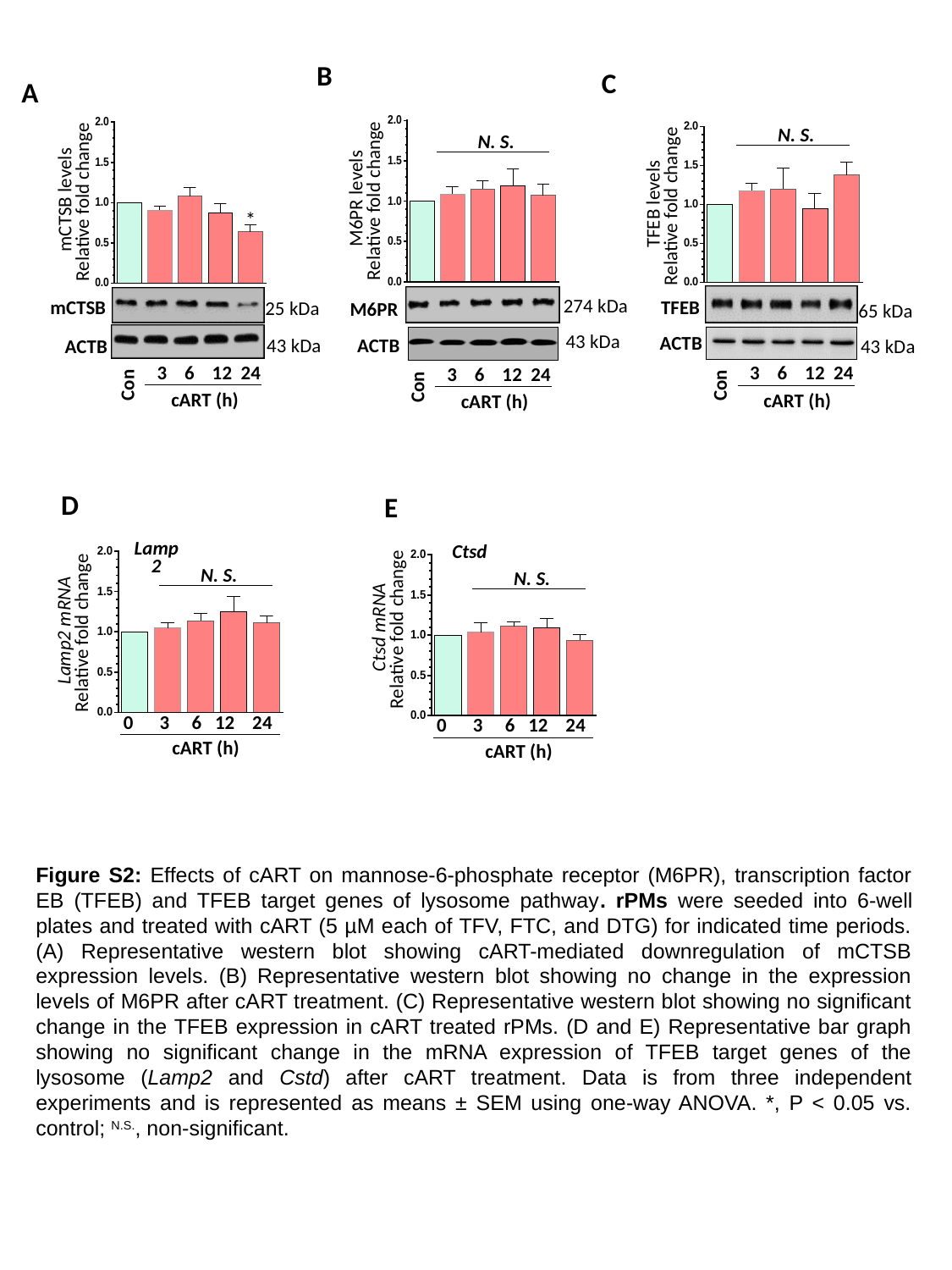

B
C
A
mCTSB levels
Relative fold change
*
M6PR levels
Relative fold change
N. S.
N. S.
TFEB levels
Relative fold change
274 kDa
TFEB
65 kDa
ACTB
43 kDa
3 6 12 24
Con
cART (h)
mCTSB
25 kDa
M6PR
43 kDa
43 kDa
ACTB
ACTB
3 6 12 24
3 6 12 24
Con
Con
cART (h)
cART (h)
D
Lamp2 mRNA
Relative fold change
Lamp2
0 3 6 12 24
cART (h)
N. S.
E
Ctsd mRNA
Relative fold change
Ctsd
0 3 6 12 24
cART (h)
N. S.
Figure S2: Effects of cART on mannose-6-phosphate receptor (M6PR), transcription factor EB (TFEB) and TFEB target genes of lysosome pathway. rPMs were seeded into 6-well plates and treated with cART (5 µM each of TFV, FTC, and DTG) for indicated time periods. (A) Representative western blot showing cART-mediated downregulation of mCTSB expression levels. (B) Representative western blot showing no change in the expression levels of M6PR after cART treatment. (C) Representative western blot showing no significant change in the TFEB expression in cART treated rPMs. (D and E) Representative bar graph showing no significant change in the mRNA expression of TFEB target genes of the lysosome (Lamp2 and Cstd) after cART treatment. Data is from three independent experiments and is represented as means ± SEM using one-way ANOVA. *, P < 0.05 vs. control; N.S., non-significant.

## Slide 4
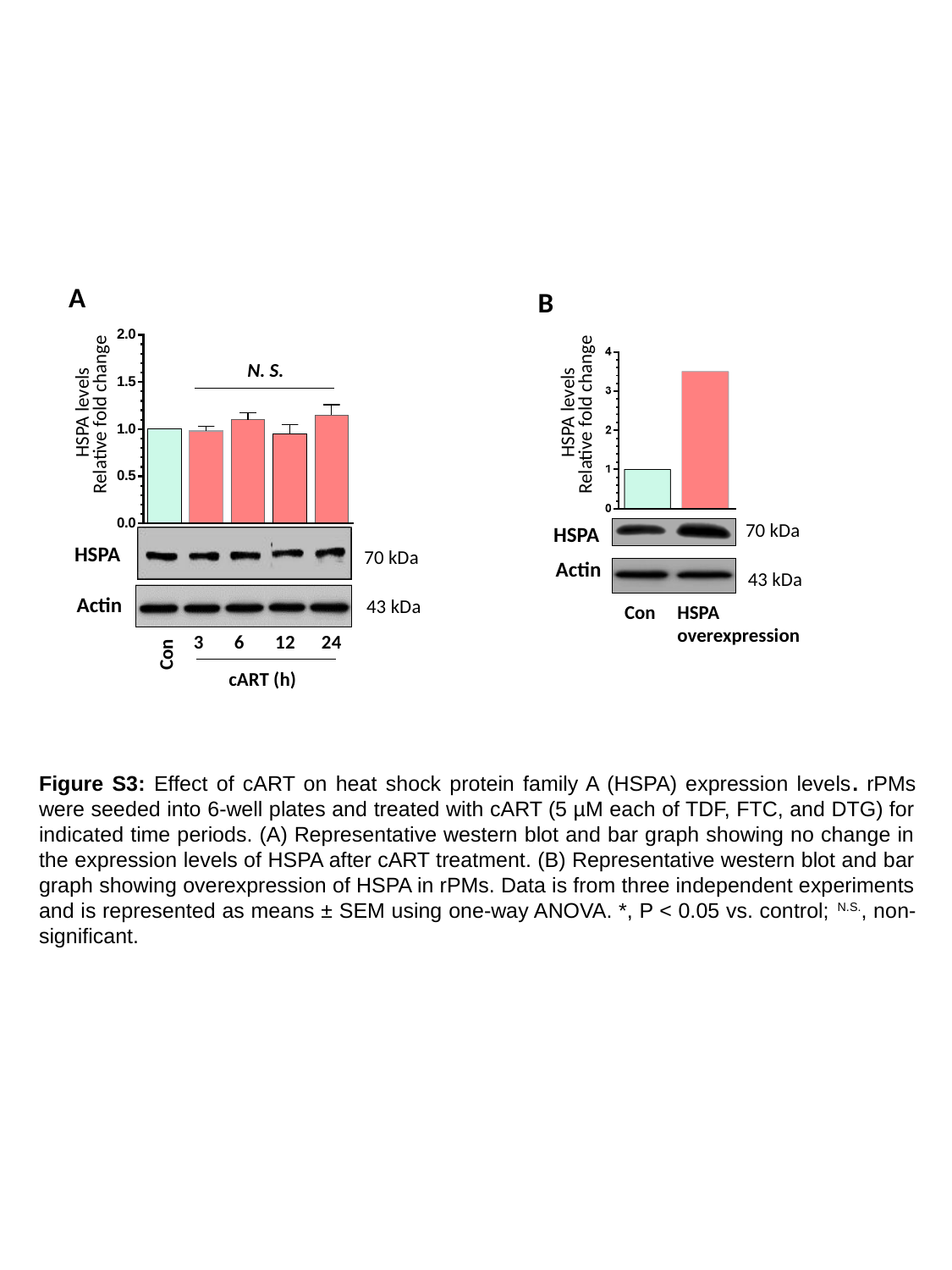

A
B
N. S.
HSPA levels
Relative fold change
HSPA levels
Relative fold change
70 kDa
HSPA
HSPA
70 kDa
Actin
43 kDa
Actin
43 kDa
Con
HSPA
overexpression
3 6 12 24
Con
cART (h)
Figure S3: Effect of cART on heat shock protein family A (HSPA) expression levels. rPMs were seeded into 6-well plates and treated with cART (5 µM each of TDF, FTC, and DTG) for indicated time periods. (A) Representative western blot and bar graph showing no change in the expression levels of HSPA after cART treatment. (B) Representative western blot and bar graph showing overexpression of HSPA in rPMs. Data is from three independent experiments and is represented as means ± SEM using one-way ANOVA. *, P < 0.05 vs. control; N.S., non-significant.
